# Case of Poisoning by the Berries of the Swamp Dogwood

**Published:** 1850-11

**Authors:** W. W. Johnson

**Affiliations:** Student of Medicine


*Case of poisoning by the berries of the Swamp Dogwood.* Reported by W. W. Johnson, N. C., Student of Medicine.

August 17th, the writer received an urgent message to visit ——— aged 4 years, who was reported dying. Found the patient in an alarming condition; there seemed to be a total deprivation of muscular power; deep and extreme coma; cold extremities; pulse weak and flickering; insensibility to questions and pinches; dilatation of pupil, and the iris so insensible that a candle held in close proximity to it did not affect it; in fact there seemed to be a general paralysis of the entire system, both nervous and muscular.

Three hours before I saw him he was attacked with a most obstinate vomiting, and while he could talk complained of pain in stomach and bowels; but at time of visit did not make any complaint—he was speechless. There was considerable dilatation of the abdomen, giving a tympanitic sound upon percussion.

Seeing the patient evidently sinking, I prescribed and administered 30 gtt. of spts. ammoniæ aromaticus, which was swallowed with difficulty, but a portion passed in the stomach, and in a few minutes gave a sufficient impetus to the languishing circulation. The symptoms were so obscure that the writer was at a great loss to account for the condition of the child.

Upon inquiry of the attendants as to what the child had been eating, I was informed by its mother and others that it had eaten nothing more than its ordinary diet; but a litttle girl, some few years older than the patient, said, "Milton had been eating some berries." She could not tell me what kind, and at my request went and brought a bunch from the meadow where the child had found them, and they proved to be the swamp dogwood.

Considering the time and the already excessive vomiting, I could anticipate no benefit from an emetic, and being apprehensive that there was a quantity in the bowels, and deeming a prompt cathartic essentially requisite, I prescribed 1 gtt. of oleum tiglii, mixed with molasses and placed far back on the tongue; this was swallowed, and in an hour and a half the child so far revived as to be able to drink a portion of a decoction of senna, which, with the oil, produced in a very short time several copious alvine dejections, mixed with which was a large quantity of the berries alluded to.

Next morning the child was as well as usual, with the exception of a very marked weakness, and recovered in a few days without any relapse whatever. [We are disposed to attribute the extreme symptoms in this case to over distention rather than to any poisonous properties of the berries of the swamp dogwood, inasmuch as the fruit of the whole genus is innocuous, and in many instances used as food. This opinion would seem to be confirmed by the relief afforded by purgation.—Ed.]

